# A participatory practice study for the improvement of sub-regional health vulnerabilities: a qualitative study

**DOI:** 10.1186/s12889-022-14111-x

**Published:** 2022-09-07

**Authors:** Jeehee Pyo, Haneul Lee, Yangwha Kang, Jaewook Oh, Minsu Ock

**Affiliations:** 1 Task Forces to Support Public Health and Medical Services in Ulsan Metropolitan City, Ulsan, Republic of Korea; 2 Department of Preventive Medicine, Asan Medical Institute of Convergence Science and Technology, University of Ulsan College of Medicine, Seoul, Republic of Korea; 3 Department of Preventive Medicine, Ulsan University Hospital, University of Ulsan College of Medicine, Ulsan, Republic of Korea; 4 Department of Counseling Psychology, Korea Counseling Graduate University, Seoul, Republic of Korea; 5 Gyeongnam Regional Center for Disease Control and Prevention, Busan, Republic of Korea; 6 Department of Preventive Medicine, University of Ulsan College of Medicine, Seoul, Republic of Korea

**Keywords:** Environment and public health, Qualitative research, Photovoice, Health disparity, Minority and vulnerable populations

## Abstract

**Background:**

This study aimed to explore the experiences of the residents of Samho-dong with the health environment in the local community, and their in-depth opinions on health promotion using a photovoice methodology. Alternatives to improve health among the residents of Samho-dong were also discussed with the local residents, with the aim of translating suggestions from the discussion into practice.

**Methods:**

A total of 195 photographs taken by the 15 participants over the course of 7 weeks were collected, along with 96 photovoice activity logs and transcription data from 5 rounds of focus group discussions. The photovoice activity logs consisted of the photographer’s name, the dates photos were taken, and a series of responses to the following SHOWeD questions: “What do you SEE here?”, “What is really HAPPENING?”, “How does this situation or scenario affect OUR lives/health?”, “WHY does this problem or strength Exist?”, “What can we DO about it?”. Direct content analysis was used for analysis.

**Results:**

The analysis yielded a total of 247 semantic units, which were categorized into the themes, “the good, but insufficiency, living environment in Samho-dong,” “the health environment in Samho-dong needs improvement,” “small efforts to improve Samho-dong,” and “points of improvement for a better Samho-dong”. Samho-dong was found to have a poorer walking and transportation infrastructure than other regions, even though it was a town with a large elderly population. The dark streets in the residential complex made participants hesitate to engage in afternoon activities, and the insufficient traffic environment made it difficult to live a natural daily life by solving food, clothing, and shelter. Participants have made various attempts to solve areas that need improvement in the Samho-dong, which has led to actual improvement. It was analyzed that in order to make Samho-dong better, it was necessary to improve the perception of residents in Samho-dong and cooperate with the local community.

**Conclusions:**

This study was significant in that it enabled the in-depth exploration and identification of areas of improvement from the participants’ perception of their health environment, considering that as residents, they are the direct stakeholders of the community health environment.

**Supplementary Information:**

The online version contains supplementary material available at 10.1186/s12889-022-14111-x.

## Background

Social interventions are needed to improve individual health, in addition to individual effort [1, 2]. Thus, the importance of a multi-level approach to health promotion and the role of local and civil society has been highlighted [3, 4]. Recently, the participation and role of members of society has been emphasized as a strategy to improve health and reduce health inequality in Republic of Korea (hereafter referred to as Korea), which have also been included in policies [5–8].

Although there are some differences in the level and degree of participation in community health promotion, Arnstein [9] and Brager and Specht [10] suggested that a higher level of participation, in which members of the community collaborate in the decision-making process as partners and exercise their decision-making power, leads to a greater potential for progressive change. In this regard, photovoice methodology, a form of participatory action research (PAR) and community-based participatory research (CBPR) may be useful for community-based health projects. Photovoice methodology is a concept that has been extended from photo-novellas and induces social change by expressing and actionizing research participants’ perceptions and experiences through the visual medium of photography [11, 12]. It has mainly been used to highlight the perspectives and experiences of vulnerable people, such as minorities and vulnerable populations [13].

Meanwhile, although Ulsan Metropolitan City (hereafter referred to as Ulsan), which is known for being the largest industrial city in Korea, experienced rapid economic growth as a result of past economic development projects, it has paid insufficient attention to health and is now faced with health indicators that are lower than those observed in other regions [14, 15]. Health discrepancies in Ulsan were also prevalent within its sub-region. Samho-dong, located in Nam-gu, Ulsan, has a higher proportion of vulnerable groups compared to other areas within the region [16]. An analysis of cumulative data from a community health survey from the past 6 years demonstrated poor statistics pertaining to walking, experience of depression, and diagnosis rates of hypertension and diabetes [17, 18]. In particular, there was a generally low prevalence of positive attitudes concerning Samho-dong, residents demonstrated a low level of satisfaction with mutual trust between neighbors, overall safety, living environment, public transportation, and medical services [18].

It is known that the health environment has an important effect on life expectancy and health-related quality of life, and is also important in alleviating health disparity [19–21]. Therefore, this study aimed to explore the experiences of the residents of Samho-dong with the health environment in the local community, and their in-depth opinions on health promotion using a photovoice methodology. Furthermore, alternatives to improve health among the residents of Samho-dong were discussed with the local residents, with the aim of translating suggestions from the discussion into practice. Until now, there have been insufficient studies in Korea to recognize residents’ perceptions of the health environment. To investigate projects to alleviate the health disparity in small areas and residents’ perceptions of the health environment of residents using photo voices. This study can be referred to as an alternative to collect opinions from local residents and strengthen their capabilities to reduce the health disparity in small areas.

## Methods

Since 2020, a pilot project is being carried out in four regions, including Nam-gu, Ulsan Metropolitan City, to establish a standard model to resolve the health disparity in small areas in Korea. This study introduced some of the contents of fostering health leaders. In the process of training health leaders, focus group discussions using photo voices were performed, and awareness and experience of the health environment were identified.

### Photovoice research

“Photovoice” is a portmanteau combining the words “photo” and “voice” and is a suitable research methodology for highlighting the experiences and perspectives of vulnerable people in alignment with its mission of “creating space for the voices of the participants” [22]. Photovoice serves to reveal the needs of the study participants through their photographic records and use them as a communication tool, drawing light to their opinions and leading to policy improvement [11, 23]. Typically, in photovoice, individuals from the local community actively participate in the entire research process (8 steps: identification → invitation → education → documentation → narration → ideation → presentation → confirmation), from the research design to establishing strategies to applying their opinions into practice [22]. This study was carried out in accordance with this eight-step process wherever possible. Furthermore, the detailed research methodology is described below according to the Consolidated Criteria for Reporting Qualitative Research (COREQ), one of the reporting guidelines for qualitative research [24].

### Selection of research participants

The research participants were recruited through collaborative efforts with the Ulsan Nam-gu Public Health Center. The Ulsan Nam-gu Public Health Center distributed guides for this study to public institutions in Samho-dong (such as the Samho-dong Community Service Center) and recruited research participants on a first-come, first-served basis. The inclusion criteria are as follows. First, a resident of Samho-dong, Ulsan, and second, voluntarily consenting to participation and photo disclosure upon receiving an explanation of the purpose and content of the study by the researcher. Twenty-two individuals applied to participate within the initial deadline, and 16 confirmed their intention to participate during a reconfirmation process following the announcement of the study schedule. Of the 16 individuals who agreed to participate, one person withdrew from the study because of personal circumstances. Thus, there were a total of 15 final participants. The main characteristics of the study participants are as listed on Table 1.

**Table 1:** Demographic information of study participants

Participant	Sex	Age (years)	Duration of residence in Samho-dong (years)
1	Female	58	31
2	Female	59	17
3	Female	63	10
4	Female	53	27
5	Female	48	25
6	Female	56	8
7	Male	63	10
8	Female	59	31
9	Female	50	5
10	Female	64	31
11	Female	60	30
12	Female	59	35
13	Female	69	15
14	Female	60	33
15	Female	60	10

### Data collection

In this study, 195 photographs taken by the 15 participants over the course of 7 weeks were collected, along with 96 photovoice activity logs and transcription data from 5 rounds of focus group discussions (FGDs). At this time, the researcher distributed “Photography Guide” leaflets to participants in preparation for unexpected situations that may arise during the data collection process. This leaflet contained a brief introduction of this study, the purpose of photography, photography instructions, and the researchers’ mobile phone number (Additional file 1). The photovoice activity logs consisted of the photographer’s name, the dates photos were taken, and a series of responses to the following SHOWeD questions [25]: “What do you SEE here?”, “What is really HAPPENING?”, “How does this situation or scenario affect OUR lives/health?”, “WHY does this problem or strength Exist?”, “What can we DO about it?” (Additional file 2).

Prior to hosting the five rounds of FGD, two instructional sessions involving an introduction of the project to resolve sub-regional health discrepancies in Samho-dong, an explanation of the photovoice and topic selection, and a photography skill training session by an expert photographer were conducted (Table 2). Following the instructional sessions, the five rounds of FGD sessions were held over 7 weeks, and each session took approximately 1 to 2 hours (Additional file 3). In the process of proceeding with FGD, the researcher who led the FGD hard to ensure that all participants had a chance to speak. In order to collect additional opinions, the researcher tried to check their stories that were not mentioned in FGD by using the photovoice activity logs. The final exhibition presented data from the photographs taken by the participants, transcripts from the five rounds of FGD, and photovoice activity logs. A detailed discussion of measures to improve the situation in Samho-dong with the study participants and public health officials followed.

**Table 2:** Summary of the study process

Session	Date	Purpose	Other
1	September 18, 2021	Introduction to the sub-regional improvement project, overview of photovoice	In-person
2	October 6, 2021	Selection of topics, introduction of preliminary research involving photovoice, photography training	In-person
3	October 7 ~ October 16, 2021	First FGD	In-person
4	October 17 ~ October 23, 2021	Second FGD	In-person
5	October 24 ~ November 6, 2021	Third FGD	In-person
6	November 5 ~ November 20, 2021	Fourth FGD	In-person
7	November 21 ~ November 27, 2021	Fifth FGD	Virtual
8	December 4, 2021	Exhibition and presentation, discussions	Virtual

### Data analysis

Data analysis within photovoice falls under “Ideation” and often involves other qualitative analysis methods [26, 27]. For this study, direct content analysis, a qualitative research method, was used for analysis [28]. Data sources were analyzed according to the weekly themed SHOWeD questions from the FGDs and activity logs, which became the basis for deductive categorization of direct content analysis.

More specifically, the researcher who led the FGD read the transcribed data from the five FGD sessions and photovoice activity logs multiple times to extract meaningful phrases on the participants’ experiences that fit the respective SHOWeD question and conceptualized them. Afterwards, the extracted concepts were reviewed by two researchers who had participated in all eight steps of the research process and the concepts were categorized according to topic and the respective SHOWeD question.

### Validity

The validity of the study was established by identifying the credibility, transferability, dependability, and confirmability proposed by Guba and Lincoln [29]. To establish credibility, the researcher shared the conceptual categorization with all 15 participants to verify that the analysis clearly reflected their experiences. For transferability, the researcher established triangulation of the data and theoretical saturation in which new data no longer appeared, by collecting various types of data through the FGDs, collection of photovoice activity logs, and collection of photographs. Furthermore, the analysis results were shared with two individuals who did not participate in the study but fit the inclusion criteria for review on whether the findings aligned with their experiences. The entire data collection and analysis process were described in detail to ensure dependability. In the process of preparing for this study, the research team became aware of the health-related environment in Samho-dong, where participants live, was weak. As this is a factor that can impact the neutrality of the study, the researchers constantly employed bracketing as a strategy to keep the knowledge of the participants’ health related to their residential environment from affecting the research process (data collection, analysis, discussion). Specifically, the researchers acknowledged their pre-understanding and discussed it together.

## Results

The analysis yielded a total of 247 semantic units, which were categorized into the themes, “the good, but insufficiency, living environment in Samho-dong,” “the health environment in Samho-dong needs improvement,” “small efforts to improve Samho-dong,” and “points of improvement for a better Samho-dong” (Table 3). Below, the participants’ experiences for each category are described.

**Table 3:** Findings per category

Themes	Sub-themes
1. The good, but insufficiency, living environment in Samho-dong	1–1. The main residents are seniors
	1–2. Restrictions in transportation due to poor walking and traffic environment
	1–3. The abundance of detached house makes it ideal for gardening
	1–4. Having parks and a river nearby make exercise more accessible
2. The health environment in Samho-dong needs improvement	2–1. Feelings of anxiety lead to being withdrawn from outdoor activities
	2–2. Poor traffic environment is a cause for inconvenience for the residents
	2–3. Residents who require assistance are alone
3. Small efforts to improve Samho-dong	3–1. Taking initiative for my own and others’ health
	3–2. Local community efforts to improve the health environment in Samho-dong
4. Points of improvement for a better Samho-dong	4–1. Residents must be considerate and make an effort to solve resident-level problems
	4–2. A health environment suitable for the residential characteristics must be established

### 1. The good, but insufficiency, living environment in Samho-dong

According to the participants' experience, Samho-dong is well suited for self-sufficiency by growing a vegetable garden and good for exercising, is certainly a good living environment for older people who are the main residents. Nonetheless, dark streets within the residential complex made the participants hesitant in engaging in afternoon activities and the poor transportation infrastructure made it difficult to retain a normal lifestyle in terms of food, clothing, and shelter.

### 1-1. The main residents are seniors

All participants agreed that Samho-dong had an aging population. One participant claimed that it had been a long time since children had been seen around the neighborhood. Some participants expressed disappointment that there were so few children, given that Samho-dong is a good environment for children, with the absence of an entertainment district and the presence of good playgrounds.

“As the population continues to age in Samho-dong, there are very few pregnant women. There are not many children, so there are very few elementary schools.” (Participant 2)

“The first thing that came to mind when I heard the term ‘vulnerable population’ was that the majority of the residents in this area are seniors. Especially since there are a lot of people spending time in Park A…” (Participant 13).

### 1-2. Restrictions in transportation due to poor walking and transportation infrastructure

**Fig. 1:**
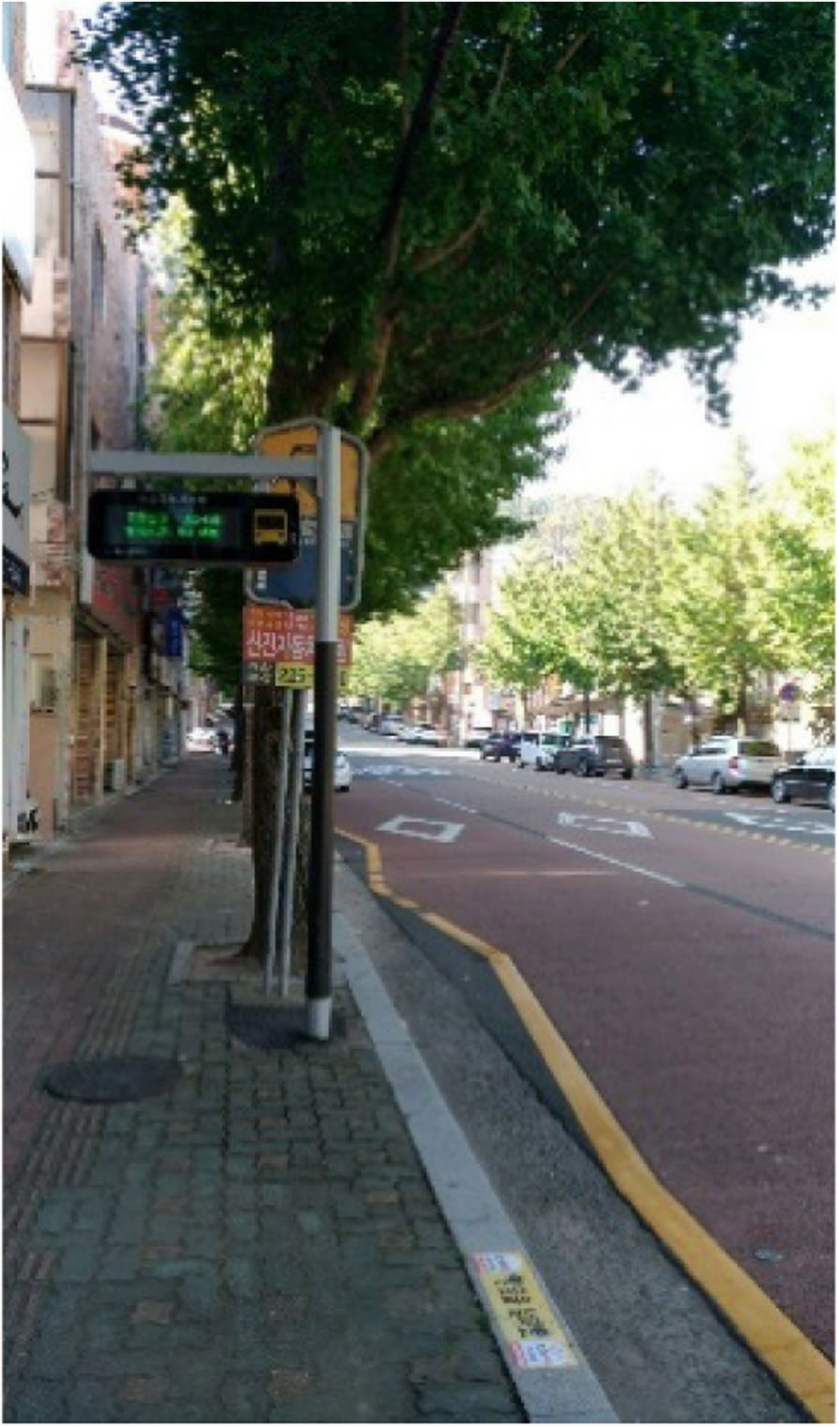
Poor bus stop conditions (Participant 12)

**Fig. 2:**
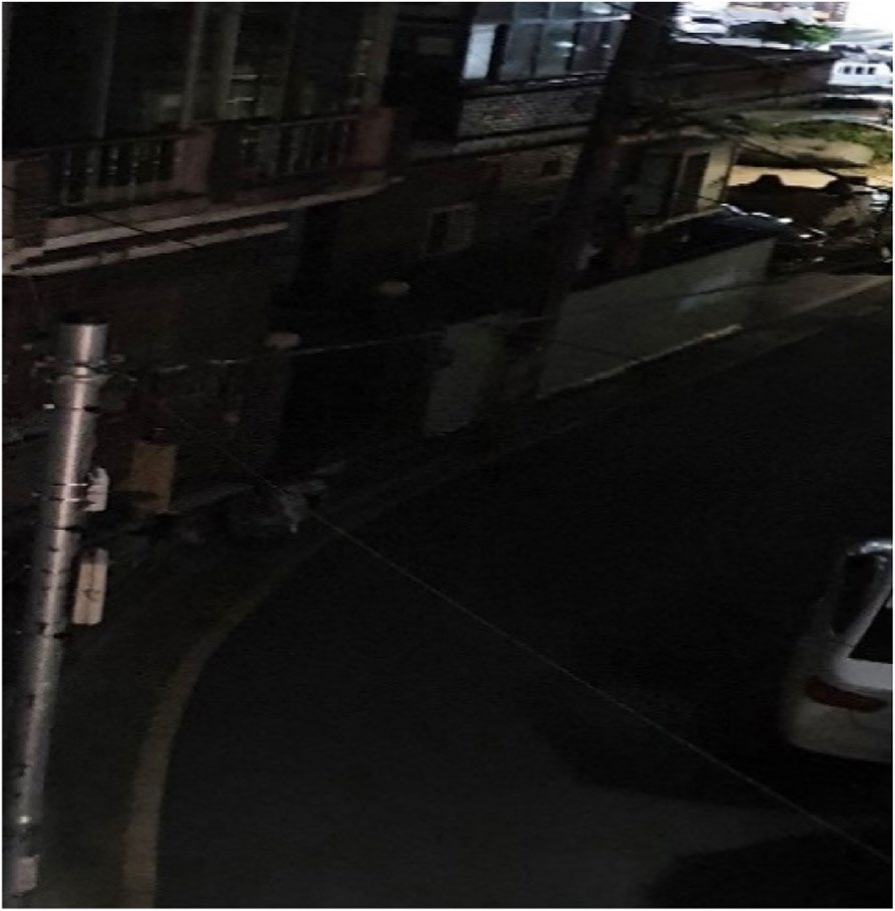
A dark street (Participant 8)

The poor transportation infrastructure in Samho-dong despite its large elderly population, is a weakness that cannot go unnoticed (Fig. 1). Difficulty in finding parking spaces and narrow and dark roads was a big problem for all residents (Fig. 2). The infrastructure under which bussing occurred, which is mainly used by the elderly, is deplorable. One participant explained that there are not many bus routes in Samho-dong, not to mention that no seating is available in bus stops despite long wait times, which forces residents to sit on the ground while waiting. Other participants claimed that although some bus stops had seating available, the seats are uncomfortable, and the bus stops environment is inadequate considering the large elderly population.

### 1-3. The abundance of detached house makes it ideal for gardening

**Fig. 3:**
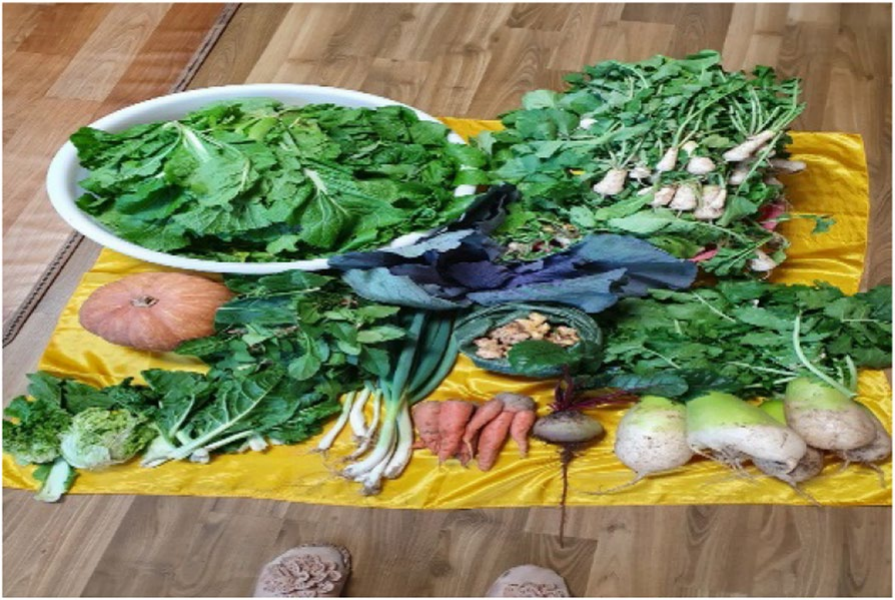
A photograph of organic vegetables grown in the garden (Participant 6)

The residential infrastructure in Samho-dong is suitable for growing vegetable gardens and serves as one factor that inspired residents to stay in Samho-dong (Fig. 3). Most participants had a vegetable garden on their roof top or in the front of their house. One participant expressed satisfaction in being able to self-produce seasonal vegetables.

“There are many residents who garden in Samho-dong thanks to the abundance of land. There are also many elderly women. This might be why there are many people who are self-sustainable. Since there are many seniors. We are doing that as well.” (Participant 2)

### 1-4. Having parks and a river nearby make exercise more accessible

**Fig. 4:**
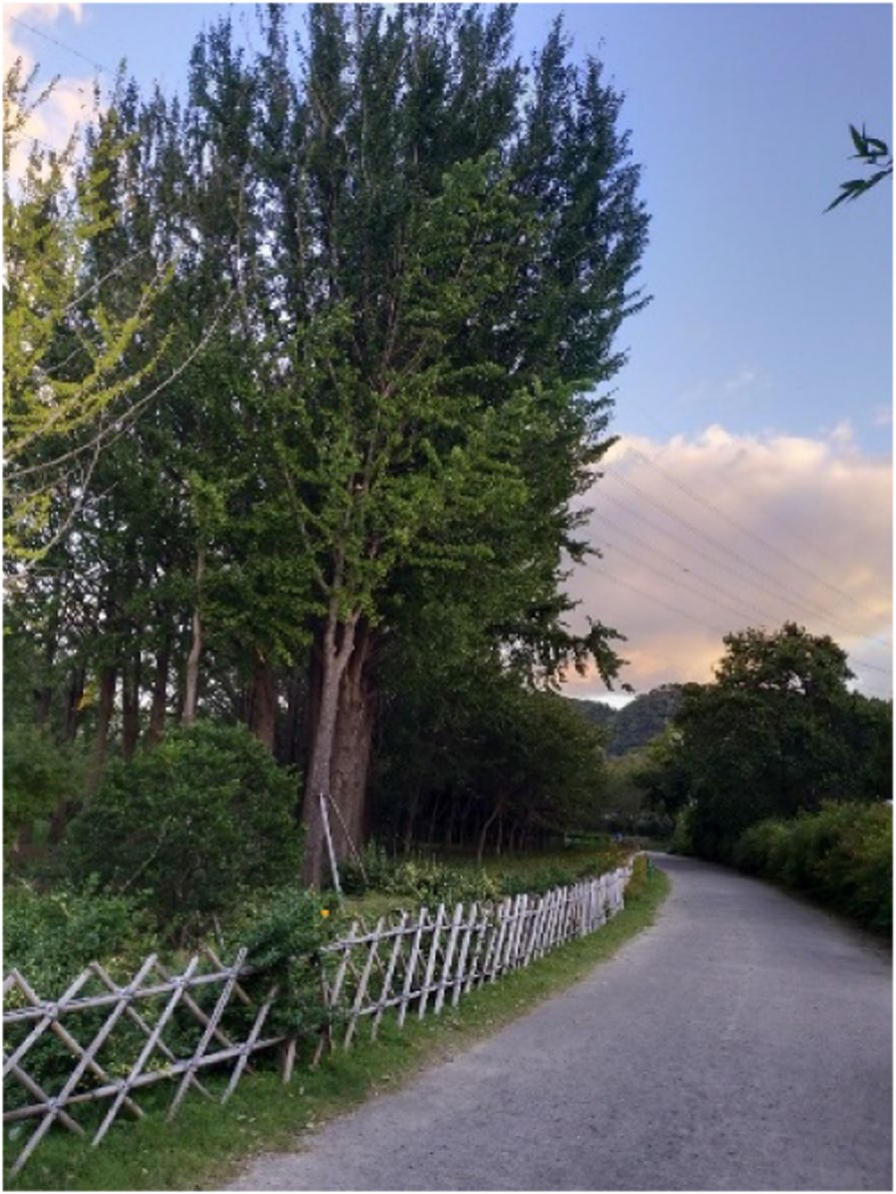
A photograph of a park in Samho-dong (Participant 4)

The presence of multiple parks and nearby rivers and mountains was a local pride of Samho-dong. The fact that there were so many good walking trails was a factor in promoting the participants' health (Fig. 4). Participants have said that because there are many good trails for walking regardless of day or night, which made Samho-dong a good place to be active despite some inconveniences in the residential infrastructure.

“Although the traffic conditions in Samho-dong are inconvenient, it has parks, and nature is so beautiful and the scenery at night is beautiful. Even if it is a little dark, my body feels so light when I walk and I love it.” (Participant 12)

### 2. The health environment in Samho-dong needs improvement

The participants perceived the generally dark streets of Samho-dong and poor transportation infrastructure as factors hindering the influx of younger people into the area. A sense of loneliness was prevalent in Samho-dong, where the majority of the residents are seniors, rather than liveliness, and one could easily find seniors spending time alone.

### 2-1. Feelings of anxiety lead to withdrawal from outdoor activities

**Fig. 5:**
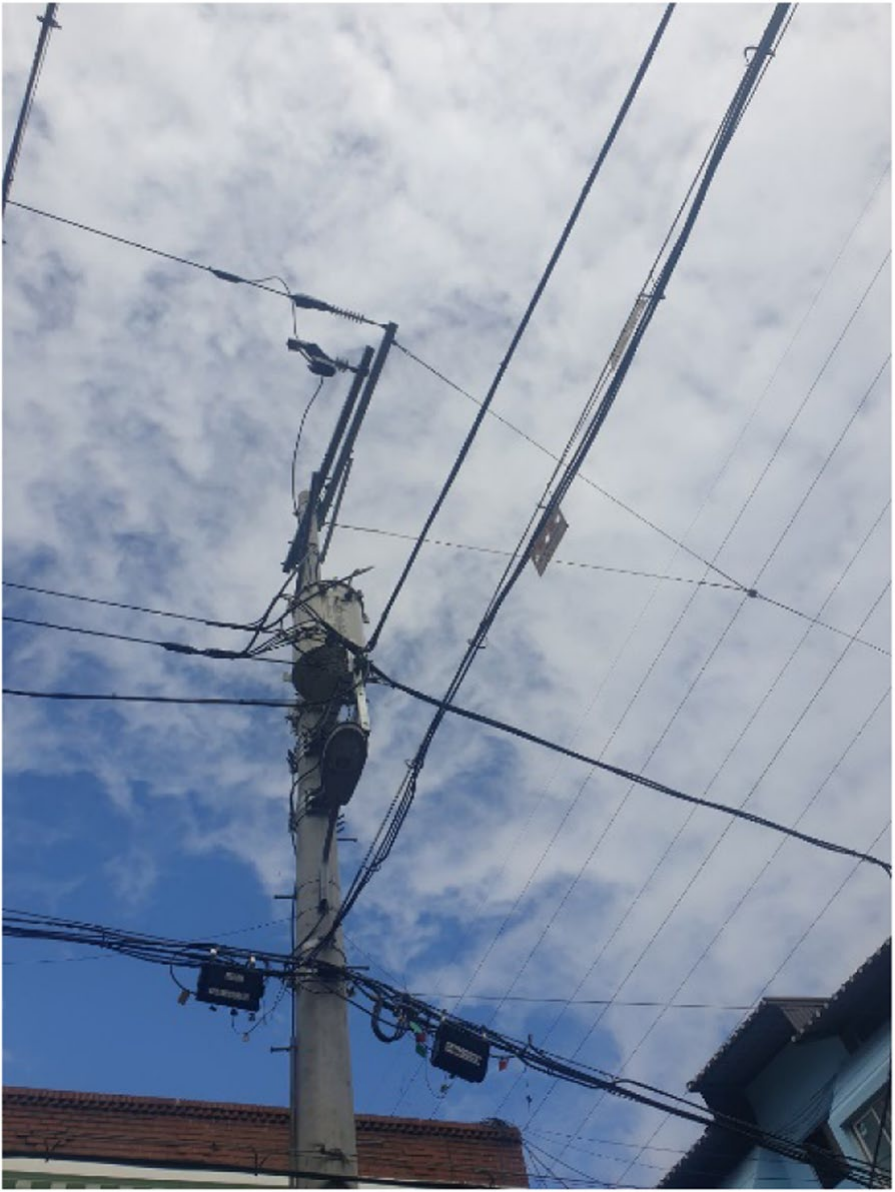
Tangled electrical wires (Participant 11)

Although ideal for growing a vegetable garden and good for exercising, there were many limitations in Samho-dong as a residential environment. Participants claimed that they felt anxious leaving their homes because of the dark streets and tangled wires. It is said that the streets are dark because the streetlights are turned off in time for the population of migratory birds, which Samho-dong is known for, to sleep, and the height of the streetlights themselves are low. The dark neighborhood hindered participants’ outdoor activities and became a source for problems, such as secretly becoming a smoking area. Powerlines coming out of houses were also cited as one of the reasons participants felt anxious. One participant complained of not wanting to look up at the sky because of the tangled wires and expressed being fearful of electromagnetic waves (Fig. 5).

“There must be a lot of electromagnetic waves and is that the internet outlet over there? I hate looking at the sky because I feel like it is going to fall.” (Participant 11)

“The darkness, it’s really not a big deal during the day, but when it gets dark at night, you get negative thoughts in your head. It’s also scary to see a cat in the dark with its eyes twinkling. So even if I want to go out to exercise in the evening, I get hesitant and, in the end, everything is connected.” (Participant 13)

### 2-2. Poor transportation infrastructure is a cause for inconvenience for the residents

**Fig. 6:**
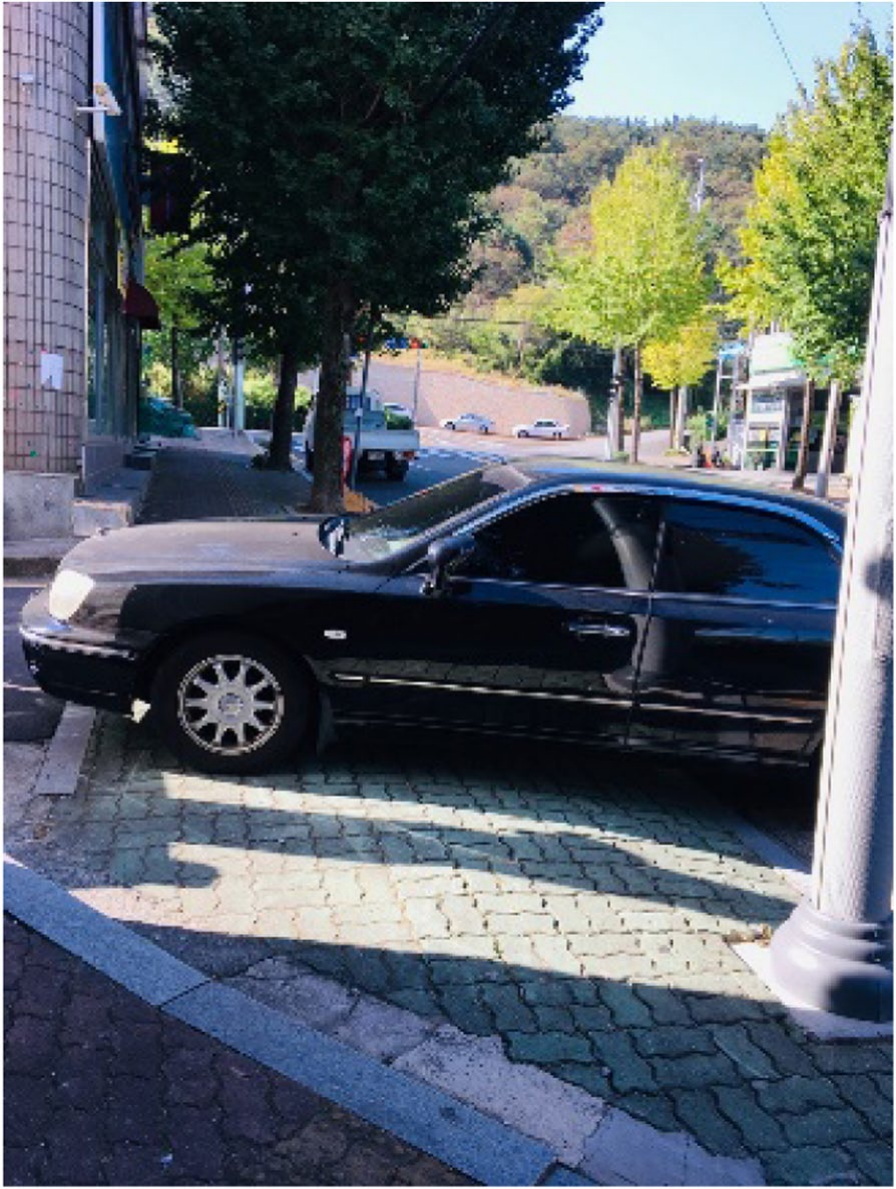
A car parked on the sidewalk (Participant 2)

Although public transit should be well equipped if parking spaces are limited and the roads are narrow, this was not the case in Samho-dong. Encountering difficulties with parking everyday caused the participants stress. Parking that blocked a portion of the sidewalk acted as barrier for pedestrians (Fig. 6).

“In the past, Samho-dong was evaluated as having good air, but now there are no young people because the traffic is so inconvenient.” (Participant 12)

**Fig. 7:**
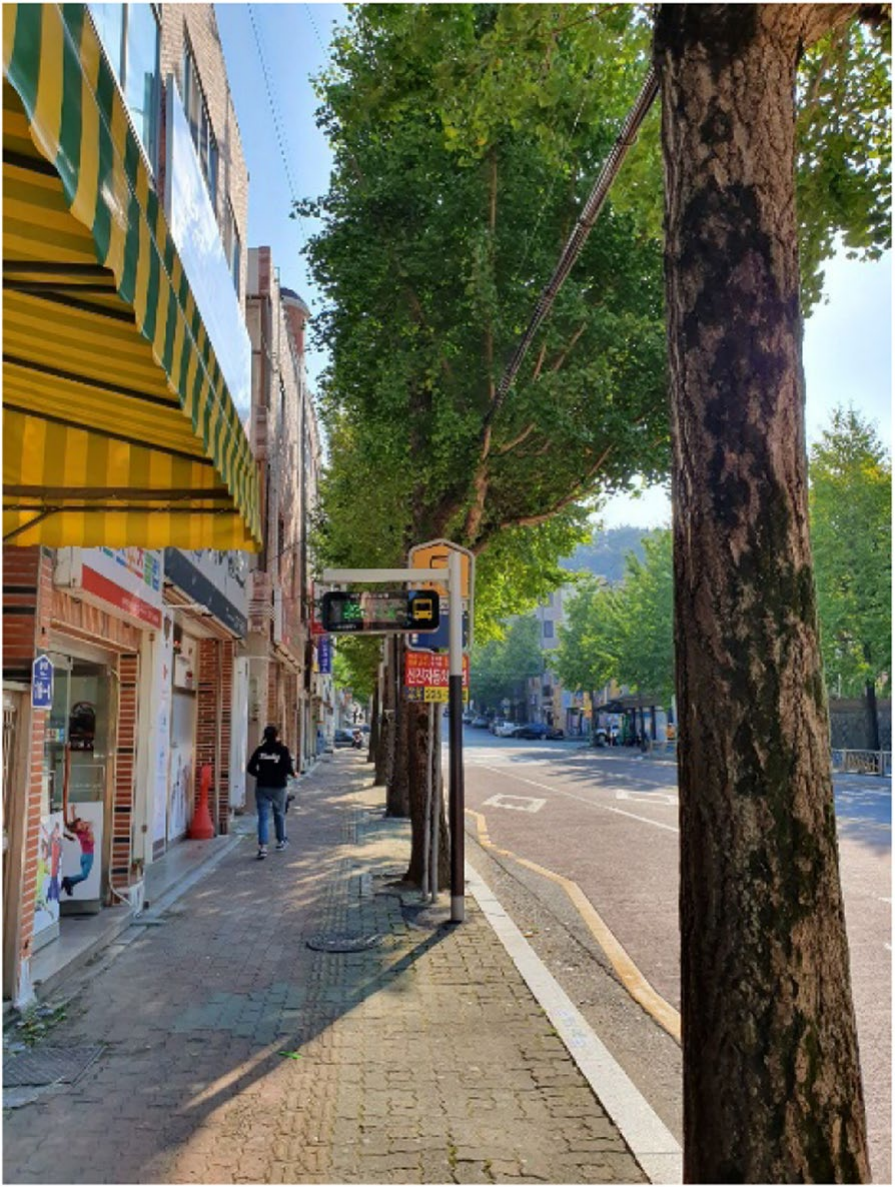
Bus stop lacking facilities (Participant 5)

The public transit conditions, which are as suboptimal as the parking issues, were said to be the reason why young people no longer stay in Samho-dong. The issues included there being only a few routes and long intervals between busses. Although bus stops must be well-equipped for waiting if intervals between busses are long, some bus stops did not even have seating (Fig. 7). In Samho-dong, where seniors who face difficulties in using smartphones make up the majority of the population, it was easy to find seniors waiting endlessly for a bus.

“Younger people can check the bus times by installing an app on their mobile phone, but seniors end up waiting for a long time because they can’t do that. They wait for a long time” (Participant 11)

“Seating, there is no seating. Elderly women sit on the roads over there on boxes. In the middle of winter, they find boxes and sit on the ground. Right now, it is a problem. Insufficient bus routes, no fans or air conditions, let alone seats, this is our neighborhood.” (Participant 15)

### 2-3. Residents who require assistance are alone

**Fig. 8:**
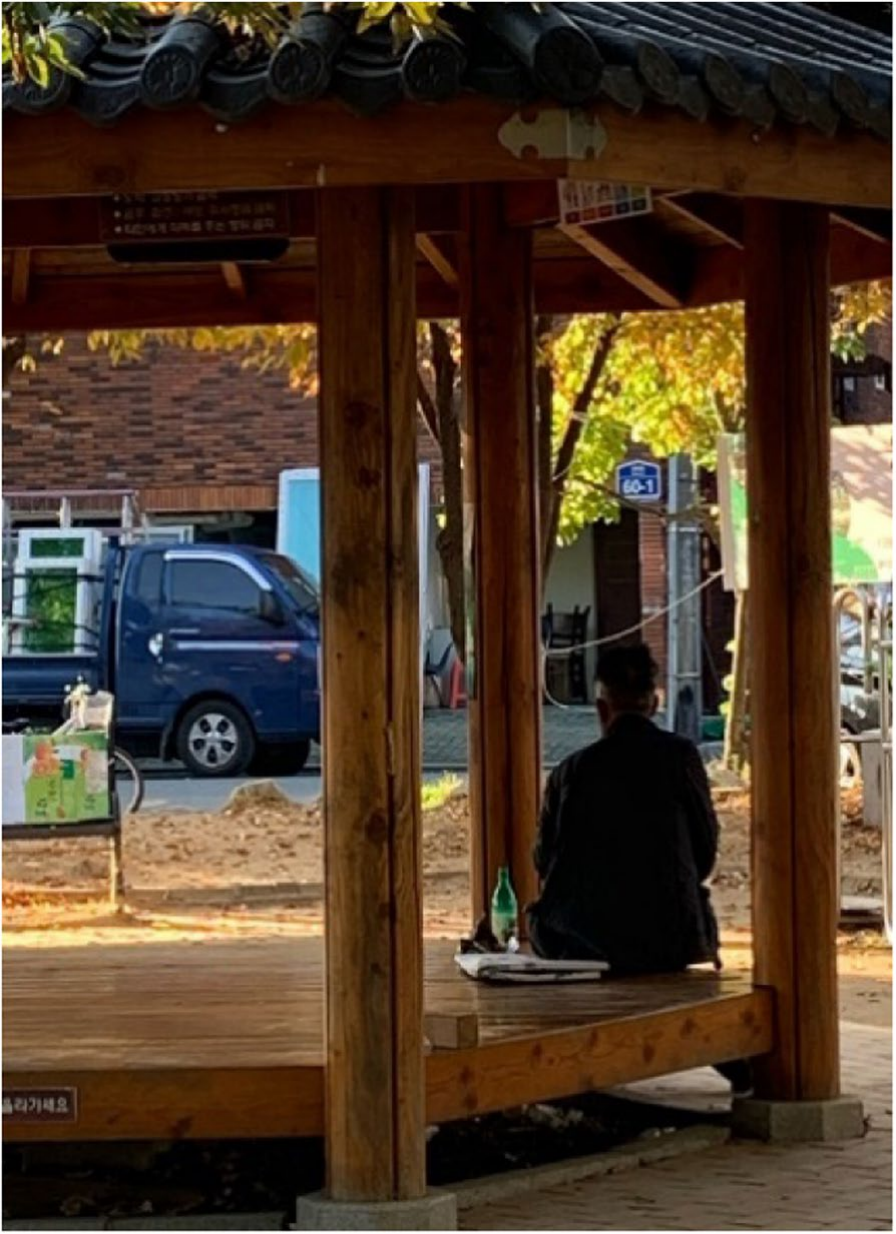
A senior sitting alone (Participant 13)

**Fig. 9:**
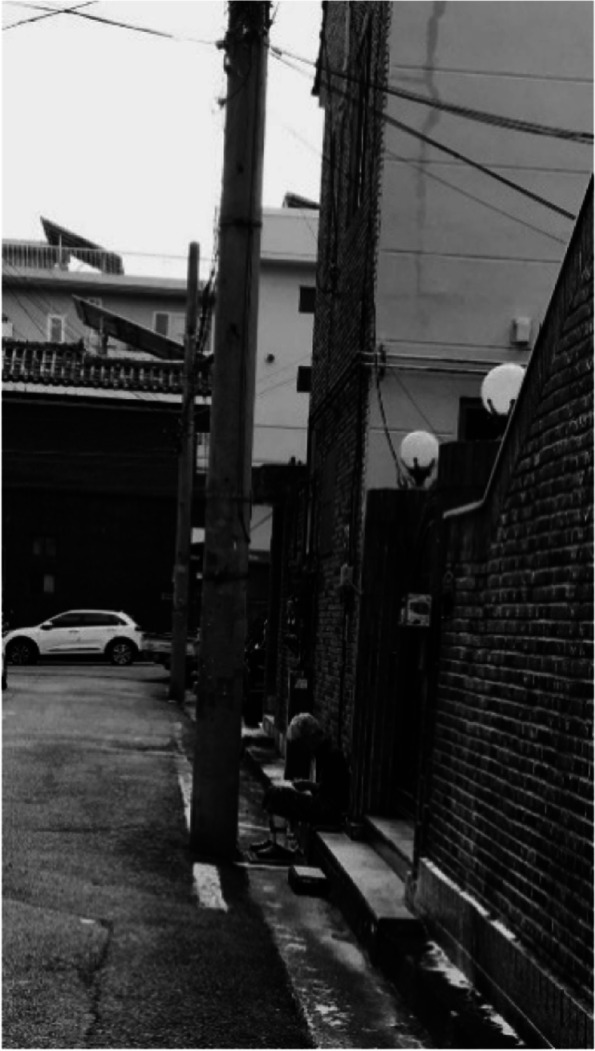
Everyday encounter with a senior with dementia (Participant 2)

It was not uncommon for participants to find seniors outside, alone (Figs. 8 and 9). Although there is a senior community center, it was overcrowded, and some stayed outside. Regardless of the season, there were many instances when seniors were observed sitting still in a park in Samho-dong. One participant expressed frustration that seniors seemed to spend their days sitting at the park with alcohol as they had no one to talk to.

“There are too many seniors, so the seniors who are outside... (omitted) cannot be accommodated in the senior community center because it is full beyond its capacity. So now that winter is here, they just sit on the chairs wearing thick clothes, observe, then return home...“ (Participant 15)

### 3. Small efforts to improve Samho-dong

Residents had a sense of ownership of Samho-dong. Thus, they made various attempts to solve issues, some of which led to actual improvements. The participants expressed discontent that the efforts of the local community did not meet the efforts of the residents.

### 3-1. Taking initiative for my own and others’ health

**Fig. 10:**
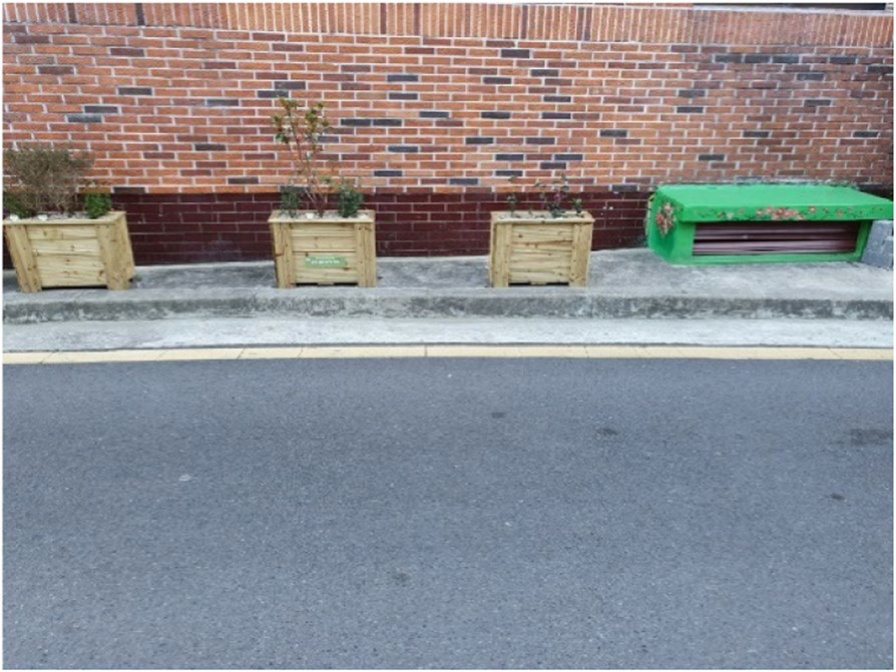
Flowerpots for improving the residential environment (Participant 6)

Participants have been actively working towards a healthy lifestyle for themselves and the residents. Most participants had taken residence-related roles, such as community leader. Such roles imposed an unspoken sense of responsibility for the neighborhood, as opposed to perceiving Samho-dong as an individual place of residence. This led to proactiveness towards serving the area. First, the participants tried to improve the local environment. They aimed to improve the residential and transportation infrastructure by regularly filing complaints to the local community, engaging in resident meetings, and installing flowerpots (Fig. 10). Looking after the vulnerable population was another responsibility taken on by the participants. They brought chairs for seniors who were sitting on the ground due to the lack of seating at bus stops. Furthermore, they actively reached out to elderly residents with dementia who were alone to ensure that they could receive support for living.

“I called her daughter to apply at the town office, but the daughter, for some reason, did not. So now she’s all alone… the helper doesn’t even come anymore. Doesn’t come and she just sits there, all alone. So now I’m her friend.” (Participant 2)

### 3-2. Local community efforts to improve the health environment in Samho-dong

Most participants felt that the local community did not take proactive measures to improve Samho-dong. Nevertheless, some participants shared experiences in improving health-related conditions in Samho-dong through the efforts of the local community. One such effort was distributing high-visibility vests to lower the risk of accidents among seniors who collect waste from early in the dawn. Another participant reflected on serving low-sodium food in the community senior welfare center, a local community effort to care for the health of seniors.

“High-visibility vests for nighttime since it’s dangerous.

I think the Ulsan metropolitan city hall checked and distributed one for all of them.” (Participant 1)

“When you go to the community senior welfare center, they serve food with very little sodium. Actually seniors hate it. Because it tastes bad. They shout, like, ‘Did you rinse the food in water or what?’ When they do that, they put salt on a plate and give it to them. Looking at it now the community senior welfare center prepares food with very little sodium.” (Participant 4).

### 4. Points of improvement for a better Samho-dong

Despite its shortcomings, Samho-dong is a valuable location where life stories are told for the participants. The participants emphasized the importance of consideration between residents and collaborative thinking and efforts of the local community in improving Samho-dong.

### 4-1. Residents must be considerate and make an effort to solve resident-level problems

**Fig. 11:**
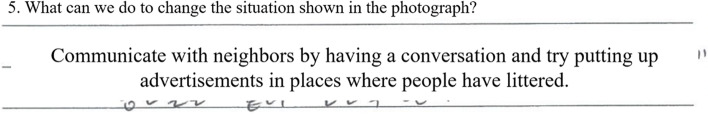
A photovoice activity log entry related to littering (Participant 4)

**Fig. 12:**
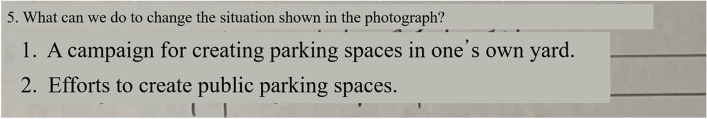
A photovoice activity log entry related to efforts to overcome parking issues (Participant 13)

To improve Samho-dong, the participants emphasized the need for an improved sense of awareness and cooperation among the residents, beyond the participants themselves. Specifically, to improve the street conditions such as leaving piles of objects and garbage in the streets, pointed out that selfish mind and attitude must change (Fig. 11). To overcome parking issues, one must make an effort to refrain from double parking and instead opt to use one’s own yard or park farther away (Fig. 12).

### 4-2. A health environment suitable for the residential characteristics must be established

In consideration of the large elderly population among the residents of Samho-dong, infrastructure improvements such as filling potholes and creating seating in bus stops must take priority. In addition, the participants expressed a wish for rest areas to be created for seniors who are out alone and for the park spaces in Samho-dong to be used for various programs designed for seniors. Participants anticipated that the effective utilization of local public resources by the local community would alleviate issues regarding the management of exercise equipment and street conditions. Participants were hopeful for residents’ health to be improved and Samho-dong to become an optimal residential neighborhood by establishing a health environment well-suited to the characteristics of Samho-dong.

“Truthfully, if anything else can be changed, it would be great if a program for seniors to entertain themselves or exercise in the park can be developed.” (Participant 11)

“Since it’ll take a long time to file a complaint to the town hall and everything, if community leaders can delegate for the purposes of volunteering or, since there are many groups in Samho-dong, if one group can manage Park A and another group works on another park, just like our own homes. Still, since we can’t fix anything broken or anything like that, the town hall should take care of that but in terms of managing, that should be enough.” (Participant 4).

## Discussion

This study examined residents’ experiences of the local health environment in Samho-dong, an area in Ulsan known for its vulnerable health conditions, and in-depth opinions on health promotion using photovoice. Instead of considering the study participants as passive research subjects, they were encouraged to participate actively in the study and think cooperatively to develop alternatives that could create a healthier environment in Samho-dong. The fact that a collaborative system has been established among the residents, along with a consensus that residential and traffic conditions must be improved alongside the health environment to alleviate the situation in Samho-dong, which involves several poor health indicators, demonstrates the practical implications of this study.

The reason for using the photovoice methodology, one of the main characteristics of this study, is as follows. First, photovoice is suitable for overcoming vulnerabilities by uncovering the experiences and perspectives of vulnerable people living in an area where the health environment is less than ideal. This study was conducted as part of a project to alleviate sub-regional health disparity by improving conditions within vulnerable areas [18], of which Samho-dong, Nam-gu, Ulsan is considered an area with a vulnerable health environment [16–18]. Second, this study aimed to have the residents of Samho-dong identify health-related vulnerabilities and develop a plan for practical improvement. The participants, who are long-term residents of Samho-dong, are aware of not only the limitations that are derived quantitatively, but also those that can only be known through experience living in the area. Using photographs taken by such participants is an effective way to reveal the real-life issues that residents face. Third, photovoice is a community-based participatory research (CBPR) method**.** That is, it does not simply identify areas for improvement, but allows findings to be reflected in policy or when developing plans [30]. Officials from the Nam-gu public health center in Ulsan were involved in the entire research process in this study and thus could recognize the need for improvement in the local community and accordingly, establish strategies. Photovoice is an optimal research method to instigate practical improvements through the inclusion of local community members as study participants and collaboration with stakeholders who can improve the community.

Recently, photovoice has been employed in various ways in health and medical research [31]. Indeed, research on various medical topics such as a study on developing a participatory community strategy for persons with disabilities [13], a participatory study on the improvement of children’s eating habits in an obesity prevention program [32], a study identifying health-related needs for women in a sub-region in Kenya [33], and a study on the management of eye health in a sub-regional perspective in India utilized photovoice [34]. In addition, in Turkey, a study has been conducted to address online education through online photovoice [35] and another study to address mental health issues during the pandemic through online photovoice [36].

On the other hand, in Korea, photovoice has been used for research on some medical topics such as obesity [37], while there is a paucity of research using photovoice in quantitative and qualitative means. This study, which, together with its residents, provided an overview of present health issues and strategies to improve the health environment in Samho-dong, may serve as a reference for future studies in the medical field using photovoice.

The participants of this study had a clear awareness of the strengths and shortfalls of the health environment in Samho-dong. First, the participants recognized the health resources in Samho-dong, namely the ideal environment for gardening and the presence of parks and a nearby river, which made it easy to stay active. Previous research findings suggest that gardening inspires healthy eating and improve one’s physical activity and ability to cope with stress [38]. Furthermore, proximity to a park is an important physical environmental factor that increase the frequency of walking [39], not to mention that simply observing nature near one’s residence can reduce stress in adults [40]. As such, making good use of the available health resources is one way to improve the health level in Samho-dong. If residents have a low level of awareness of the available health resources, a promotion strategy to increase awareness may also be needed.

On the other hand, participants identified as health-related shortfalls the low sense of vitality in the area due to the large elderly population and limited mobility due to poor walking and traffic conditions. As part of the strategy to revitalize the local community and improve accessibility, establishing infrastructure such as a ‘Wawa (와와 in Korean)’ community house or expanding the Centers for Supporting Healthy Living and developing various programs using the parks in Samho-dong may be considered.

Moreover, it may be necessary to promote a health environment that is well-suited to the characteristics of the residents in Samho-dong. Previous research findings indicate that factors such as narrow walking paths, road surface conditions, the shortage of benches, and steep inclines make walking activities difficult for seniors [39]. Considering that the majority of residents in Samho-dong are seniors, filling potholes and establishing a waiting area in bus stops may be feasible solutions. Furthermore, based on preliminary research findings that suggest that the frequency of walking increases and the lower the body mass index with greater satisfaction towards public transit [41], considering means to activate public transportation in Samho-dong, such as reducing wait times and creating new routes may be helpful.

It has been suggested that there is a relatively low rate of management of hypertension and diabetes in Samho-dong compared to other areas [17, 18]. To increase the management rate of hypertension and diabetes, it is necessary to not only expand treatment facilities, but also improve accessibility to healthy food and exercise equipment [42]. Previous research also demonstrated that the health behaviors of hypertensive and diabetic Koreans were not particularly better – sometimes even worse – than those of the general population [43]. Informing residents who have hypertension or diabetes of accessible exercise equipment and stores in which fresh produce can be easily obtained may also be important.

This study also developed a multi-level strategy for improving the health environment. To improve the health level of local community residents, establishing various health environments is important, alongside efforts at the level of the individual. Nevertheless, creating a space for residents to overcome local issues on their own may be most important [3, 4]. Together, the participants and researchers of this study discussed strategies to improve Samho-dong over the long term and it is expected that the participants will act as health leaders in Samho-dong. As such, the long-term effectiveness of a health care project can only be demonstrated if such collaborative efforts serve as a basis for change.

### Limitation

One limitation of this study is that when the improvement strategies developed through photovoice were implemented in practice, they did not lead to immediate change. Following the post-exhibition discussion, participants, public health officials and district councilors hoped to instigate practical changes by listing the vulnerabilities where improvement was necessary (Additional file 4). Nevertheless, the scope of this study did not cover the budget established to improve the identified vulnerable areas. Ultimately, the goal of participatory research using photovoice is to implement the strategies developed throughout the study in practice and lead to improvements. In the future, it will be necessary to review whether the health environment in Samho-dong has indeed improved and track the participants’ changes in self-efficacy. Another limitation of this study is that most of the participants were women. Although we did not find any particular differences in results between men and women, men and women may have different perceptions of the health environment. It will be necessary to conduct a similar study involving men in the future.

Despite such limitations, this study was significant in that it enabled the in-depth exploration and identification of areas of improvement from the participants’ perception of their health environment, considering that as residents, they are the direct stakeholders of the community health environment. Furthermore, this study had practical implications such as increasing the residents’ interest in issues pertaining to the health environment, thus increasing resident participation in the health promotion project. This study served to not only collect evidence to improve the health of residents in Samho-dong, an area with vulnerable health conditions, but also may serve as a reference for future participatory local community programs in other regions.

## Conclusions

This study was significant in that it enabled the in-depth exploration and identification of areas of improvement from the participants’ perception of their health environment, considering that as residents, they are the direct stakeholders of the community health environment. Furthermore, this study had practical implications such as increasing the residents’ interest in issues pertaining to the health environment, thus increasing resident participation in the health promotion project. This study served to not only collect evidence to improve the health of residents in Samho-dong, an area with vulnerable health conditions, but also may serve as a reference for future participatory local community programs in other regions.

## Supplementary Information

**Additional file 1. **Instruction pamphlet.

**Additional file 2.** Training log for health leadership activities.

**Additional file 3.** Process of photovoice focus group discussions.

**Additional file 4. **List of areas of improvement for practical development.

## Data Availability

All data generated or analysed during this study are included in this published article and its supplementary information files.

## Abbreviations

PAR: Participatory action research
CBPR: Community-based participatory research
FGDs: Focus group discussions
