# Supplementary figures and images for: A participatory practice study for the improvement of sub-regional health vulnerabilities: a qualitative study
Source: BMC Public Health. 2022 Sep 7;22:1698. doi: 10.1186/s12889-022-14111-x (PMC9454115; doi:10.1186/s12889-022-14111-x)

**[Appendix 1: Instruction Pamphlet]**


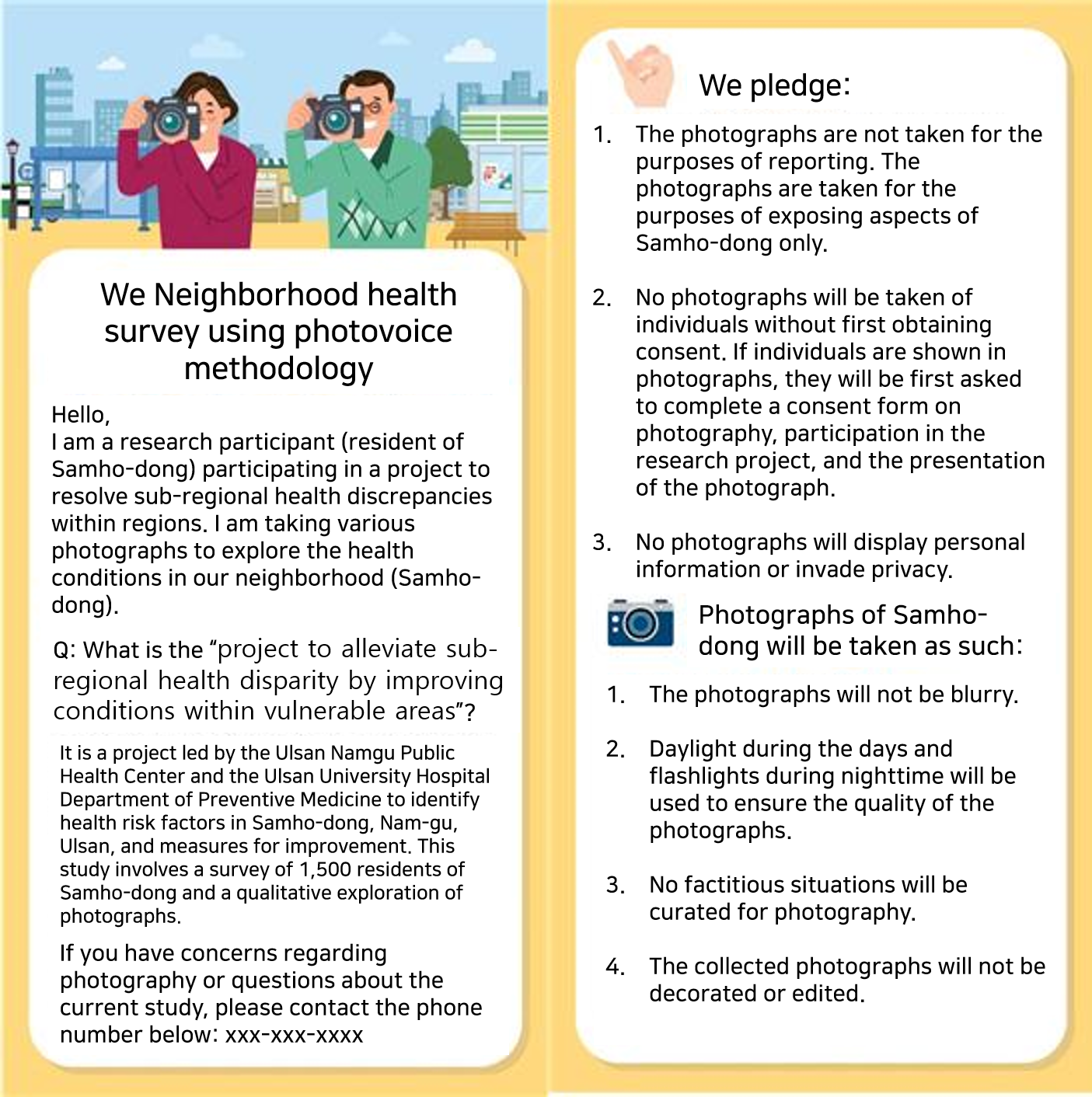

Supplement: Supplementary file 1 — Additional file 1. Instruction pamphlet. [file 12889_2022_14111_MOESM1_ESM.docx]

**[Appendix 3: Process of Photovoice Focus Group Discussions]**

**
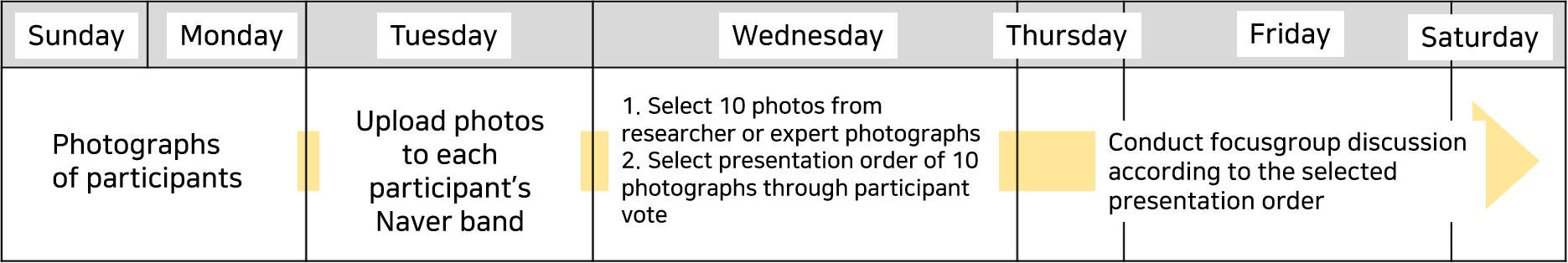
**

Supplement: Supplementary file 3 — Additional file 3. Process of photovoice focus group discussions. [file 12889_2022_14111_MOESM3_ESM.docx]
