# Supplementary material for: A participatory practice study for the improvement of sub-regional health vulnerabilities: a qualitative study
Source: BMC Public Health. 2022 Sep 7;22:1698. doi: 10.1186/s12889-022-14111-x (PMC9454115; doi:10.1186/s12889-022-14111-x)
Supplement: Supplementary file 2 — Additional file 2. Training log for health leadership activities. [file 12889_2022_14111_MOESM2_ESM.docx]

**[Appendix 2: Training Log for Health Leadership Activities]**

| Date of training |  | Method |  |
| --- | --- | --- | --- |
| Contents | Training log | | Time |
|  | This photograph of _______ was uploaded by ___________ on ____(month) ___(day)  1. What do you SEE here? (What is the first thing you notice in the photograph?)  2. What is really HAPPENING? (What is the story behind this photograph? Why was the photo taken?)  3. How does this situation or scenario affect OUR lives/health? (Who is affected? Who is not?)  4. WHY does this problem or strength exist? (How did things become this way? What caused things to be this way?)  5. What can we DO about it? | |  |
| Instructor and location | Name | | Address |
|  |  | |  |
