# Supplementary material for: A participatory practice study for the improvement of sub-regional health vulnerabilities: a qualitative study
Source: BMC Public Health. 2022 Sep 7;22:1698. doi: 10.1186/s12889-022-14111-x (PMC9454115; doi:10.1186/s12889-022-14111-x)
Supplement: Supplementary file 4 — Additional file 4. List of areas of improvement for practical development. [file 12889_2022_14111_MOESM4_ESM.docx]

**[Appendix 4: List of Areas of Improvement for Practical Development]**

| **Category** | **Suggestions for improvement** | **Number of locations** | |
| --- | --- | --- | --- |
| Street conditions | Installation of streetlights | 3 |  |
|  | Filling potholes (level out roads) | 9 |  |
|  | Removal of barriers | 8 |  |
|  | Implementation of slip-proof sidewalks | 1 | |
| Parking conditions | Management of no-parking zones | 4 | |
|  | Provision of parking spaces | 1 | |
| Bus stop conditions | Establishment of a pest control plan | 1 | |
|  | Upgrade seating in bus stops | 1 | |
|  | Update advertisements | 1 | |
| Other environmental conditions | Provision of rest areas | 4 | |
|  | Replace dead flowers and trees around neighborhood | 2 | |
|  | Maintenance and replacement of exercise equipment | 2 | |
|  | Alleviation of unpleasant odors | 2 | |
|  | Update traffic lights (visibility issue) | 1 | |
